# Early identification of refractory *Mycoplasma pneumoniae* pneumonia in children using CT-based radiomics: a multicenter study

**DOI:** 10.3389/fmed.2026.1785817

**Published:** 2026-03-19

**Authors:** Qian Li, Jian Zhang, Zi-Jun Song, Wenjing Chen

**Affiliations:** 1 Department of Critical Care Medicine, Baoding First Central Hospital, Baoding, China; 2 Department of Research and Development, United Imaging Intelligence (Beijing) Co., Ltd., Beijing, China

**Keywords:** children, machine learning, *Mycoplasma pneumonia*, radiomics, X-ray computed tomography

## Abstract

**Objective:**

To develop and validate a model that utilizing clinical, imaging, and radiomics characteristics for early predicting refractory *Mycoplasma pneumoniae* pneumonia (RMPP) in children.

**Methods:**

This multicenter retrospective study included a total of 419 children, divided into training (*n* = 248), testing (*n* = 62), and external validation (*n* = 109) cohorts. Patients were classified into non-RMPP and RMPP groups based on clinical guidelines. Radiomics features were extracted from chest CT scans using PyRadiomics, followed by SelectKBest and least absolute shrinkage and selection operator regression for feature selection. Three random forest-based predictive models were developed: clinical-imaging, radiomics, and integrated. Predictive performance was evaluated using the area under the receiver operating characteristic curve (AUC), McNemar tests, and net reclassification improvement (NRI).

**Results:**

The integrated model demonstrated the highest predictive performance (AUC: 0.811, 95% CI: 0.704–0.917), compared with both the radiomics (AUC: 0.784, 95% CI: 0.683–0.885) and clinical-imaging (AUC: 0.675, 95% CI: 0.603–0.833) models in the validation cohort. McNemar tests revealed significant differences in classification between the radiomics and clinical-imaging models (*p* = 0.001), radiomics and integrated models (*p* = 0.013), and clinical-imaging and integrated models (*p* < 0.001) in the validation cohort. In the validation cohort, the NRI was higher for the integrated model than for the radiomics and clinical-imaging models (both *p* < 0.001) but did not differ between the radiomics and clinical-imaging models (*p* = 0.070). Key predictors included D-dimer, type of fever, and the systemic immune-inflammation index, along with radiomics features such as gray-level co-occurrence matrix and wavelet kurtosis.

**Conclusion:**

The integrated model, combining clinical, imaging, and radiomics features, enhances risk stratification for RMPP.

## Introduction

*Mycoplasma pneumoniae* pneumonia (MPP) is a major pediatric public health concern worldwide, accounting for approximately 10–40% of community-acquired pneumonia cases among children aged 5–15 years (1, 2). As an atypical pathogen, *Mycoplasma pneumoniae* infection often has an insidious onset and a protracted course, which may lead to prolonged hospitalization, increased antibiotic resistance pressure, and long-term sequelae such as bronchiolitis obliterans or pulmonary fibrosis (3). Refractory MPP (RMPP) is characterized by persistent fever, radiological deterioration, and inadequate response to macrolide therapy despite appropriate treatment (typically ≥7 days) (3). This subtype affects ~5–20% of MPP cases and is associated with higher rates of complications, including pleural effusion, atelectasis, and extrapulmonary manifestations such as encephalitis or myocarditis (4, 5). With the rising incidence of RMPP driven by macrolide resistance and immune dysregulation (3, 6), early identification strategies that enable timely therapeutic optimization are urgently needed.

Imaging is central to the assessment of MPP. Early studies relied on chest radiography for initial evaluation (6), whereas computed tomography (CT) provides superior depiction of ground-glass opacities, centrilobular nodules, and bronchial wall thickening—findings correlated with disease severity (6, 7). However, conventional imaging assessment remains subjective and may lack consistency, particularly across centers, limiting its translation into robust risk scores. Radiomics has emerged as a transformative approach that extracts high-throughput quantitative features from CT images to characterize tissue heterogeneity beyond visual inspection (8–10). Prior investigations on MPP risk stratification have largely focused on clinical symptoms (11), biomarkers (3), or conventional imaging evaluations (12), yet these approaches are often constrained by subjectivity, delayed actionable information, and limited predictive accuracy (13).

Therefore, we developed and externally validated an explainable, admission-time CT-based radiomics model integrated with clinical and conventional imaging variables and quantified its incremental predictive and decision-analytic value for early identification of pediatric RMPP.

## Materials and methods

### Study setting and participants

This multicenter study adhered to the Declaration of Helsinki and received approval from the Ethics Committee of Baoding First Central Hospital (Approval No. HDFYLL-KY-2024-002), with informed consent waived due to its retrospective nature. Cases were retrospectively collected from two medical centers between July 2024 and July 2025 and randomly allocated to training and internal testing cohorts at an 8:2 ratio. Simultaneously, cases from the same timeframe were sourced from one other medical center to form an external validation cohort.

Inclusion criteria were: (1) participants aged over 1 month and under 18 years; (2) MPP confirmed according to the guideline (14), requiring pneumonia symptoms and signs plus a positive lab finding from either nucleic acid testing for MP, or MP antibody levels of ≥1:160 in a single test or a fourfold rise via serum agglutination during illness. Exclusion criteria encompassed: (1) underlying conditions such as immunodeficiency, chronic lung diseases, heart disorders, chronic kidney diseases, rheumatic conditions, malnutrition, diabetes, or genetic/metabolic disorders; (2) concurrent infections with other respiratory pathogens; (3) missing clinical information; and (4) history of lung surgery.

The index time (baseline) was defined as the time of hospital admission for MPP. To enable early prediction and avoid information leakage, all candidate predictors were obtained at baseline, defined as values recorded within the first 24 h after admission. Patients were categorized into non-RMPP and RMPP groups. RMPP was defined according to the guideline (14) as persistent fever (>38.5 °C), worsening clinical symptoms, and deteriorating radiological findings after ≥7 days of appropriate macrolide therapy. The “deteriorating radiological findings” criterion was determined based on follow-up imaging compared with the baseline CT. Patients who responded to standard treatment with clinical and radiological improvement before Day 7 were classified as non-RMPP.

### Clinical data collection and imaging analysis

Patient data were retrospectively collected, encompassing demographic details, clinical manifestations, laboratory results, and imaging features. Demographic variables included sex and age, while clinical presentation covered type of fever (absent, low-grade, mid-grade, or hyperpyrexia). Laboratory parameters comprised white blood cell (WBC) count, neutrophil (NEUT) count, platelet (PLT) count, creatine kinase-MB (CK-MB), lactate dehydrogenase (LDH), activated partial thromboplastin time (APTT), fibrinogen (FIB), D-dimer, C-reactive protein (CRP), neutrophil-to-lymphocyte ratio (NLR), platelet-to-lymphocyte ratio (PLR), and systemic immune-inflammation index (SII). The SII was computed as: PLT count × NEUT count/lymphocyte count.

All pediatric patients underwent chest CT examinations during hospitalization. For model development, the baseline CT used for radiomics feature extraction was defined as the first chest CT performed within 48 h after admission. If more than one CT scan was available within this window, the earliest scan was selected. CT scans performed after Day 7 of macrolide therapy or after fulfillment of the refractory criteria were not used for feature extraction to prevent outcome-related information leakage.

All pediatric patients underwent chest CT scans during hospitalization. Two experienced radiologists (with 6 and 8 years of diagnostic expertise, respectively) independently evaluated the images. Disagreements were resolved by consensus with a senior radiologist (12 years of experience). Radiologists remained blinded to clinical information and patient identities to reduce bias. Radiological evaluation emphasized key CT findings: lobar atelectasis, consolidation pattern, consolidation mixed with ground-glass opacity, adjacent pleural thickening, pleural effusion, mediastinal lymph node enlargement, air bronchogram sign, interlobular septal thickening, reticular pattern, fiber cord, and number of involved lobes.

### Image acquisition and preprocessing

Chest CT scans were acquired using multiple scanner models, with specifications and parameters detailed in the Supplementary Table S1. Examinations were conducted with patients in the supine position during end-inspiratory breath-holding, preceded by standardized training to optimize image quality. Scan coverage extended from the thoracic inlet to the costophrenic angles.

Preprocessing entailed resampling images to 1 mm^3^ isotropic voxels via B-spline interpolation, alongside intensity histogram normalization.

### Region of interest segmentation

Region of interest (ROI) encompassing pulmonary lesions were manually delineated on chest CT scans using ITK-SNAP (v4.0.0; http://www.itksnap.org/pmwiki/pmwiki.php) by two radiologists, with subsequent consensus and review by a senior radiologist (>15 years of experience). High inter-observer agreement was attained (inter-observer Dice 0.87 ± 0.04).

### Radiomics feature extraction, selection

Radiomics features were extracted using PyRadiomics version 3.0 (15). In total, 1,904 features were derived from original and filtered CT images through 15 filters (Supplementary material), spanning seven categories: first-order statistics (*n* = 378), shape descriptors (*n* = 14), gray-level co-occurrence matrix (GLCM; *n* = 441), gray-level run-length matrix (GLRLM; *n* = 336), gray-level size-zone matrix (GLSZM; *n* = 336), gray-level dependence matrix (GLDM; *n* = 294), and neighboring gray-tone difference matrix (NGTDM; *n* = 105).

Subsequently, the SelectKBest method was employed to retain half of the original features, followed by least absolute shrinkage and selection operator (LASSO) regression to address multicollinearity. To strictly avoid information leakage, feature selection and all preprocessing steps were conducted exclusively on the training cohort. The selected features were subsequently applied unchanged to the external validation cohorts. Default scikit-learn hyperparameters were used for the random forest models, with no additional tuning.

### Model development

**Figure 1:**
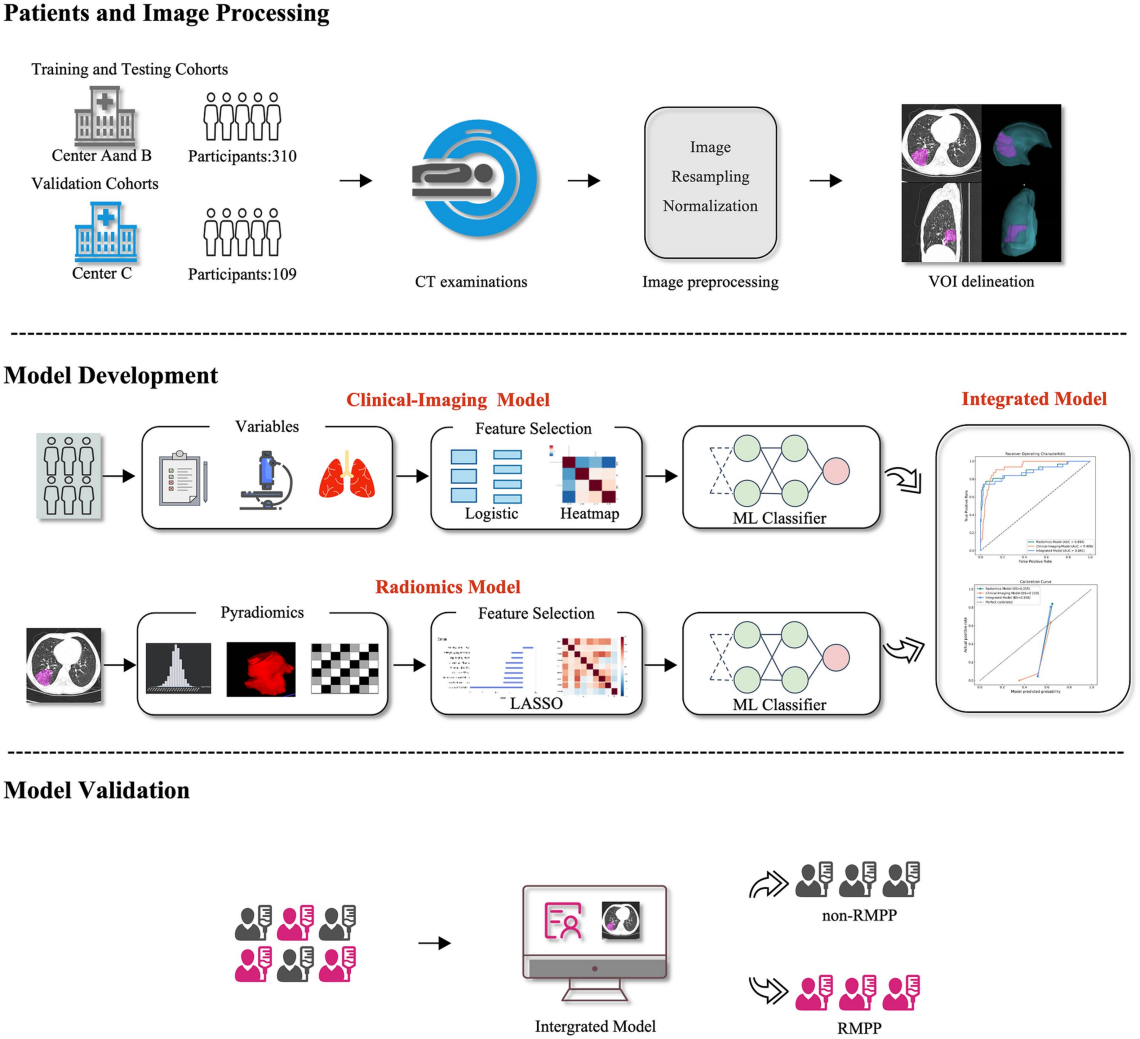
Flow chart of the study. CT, computed tomography; VOI, volume of interest; ML, machine learning; LASSO, least absolute shrinkage and selection operator; Non-RMPP, non-refractory *Mycoplasma pneumoniae* pneumonia; RMPP, refractory *Mycoplasma pneumoniae* pneumonia.

For the clinical-imaging model, univariable logistic regression assessed associations between clinical and imaging variables and RMPP. Variables achieving *p* < 0.05 were deemed significant and incorporated into subsequent modeling. Three random forest models were constructed: a clinical-imaging model utilizing clinical and imaging variables, a radiomics model relying solely on selected radiomics features, and an integrated model fusing clinical, imaging, and radiomics features. Figure 1 presents a schematic overview of the system.

### Statistical analysis

Continuous variables are presented as medians with interquartile ranges (IQRs), and categorical variables as counts with percentages. Patient characteristics were compared among training, testing, and validation cohorts using Fisher’s exact test, Pearson’s *χ*^2^ test, or Mann–Whitney *U* test, selected based on data type and distribution. Univariable logistic regression was employed to evaluate associations between clinical and imaging variables and RMPP.

Model performance was assessed via receiver operating characteristic (ROC) curves and areas under the curve (AUC) with 95% confidence intervals. Supplementary metrics—sensitivity, specificity, accuracy, *F*_1_-score, and precision—were computed for thorough evaluation. Inter-model comparisons employed DeLong’s test, McNemar’s test, net reclassification improvement (NRI), and integrated discrimination improvement (IDI). To enhance interpretability of the Integrated model, we performed SHapley Additive Explanations (SHAP) analysis to evaluate the relative contribution of each feature to the model’s output.

All statistical analyses were conducted in R (version 3.6.0; R Foundation for Statistical Computing), whereas machine-learning model construction and performance evaluation were implemented in Python using scikit-learn (sklearn, version 1.4.2), with two-sided *p* < 0.05 indicating statistical significance. The classification threshold for all random forest models was fixed at the default probability of 0.5, which was pre-specified during training and frozen before external validation to prevent any data leakage.

## Results

### Patient characteristics

**Figure 2:**
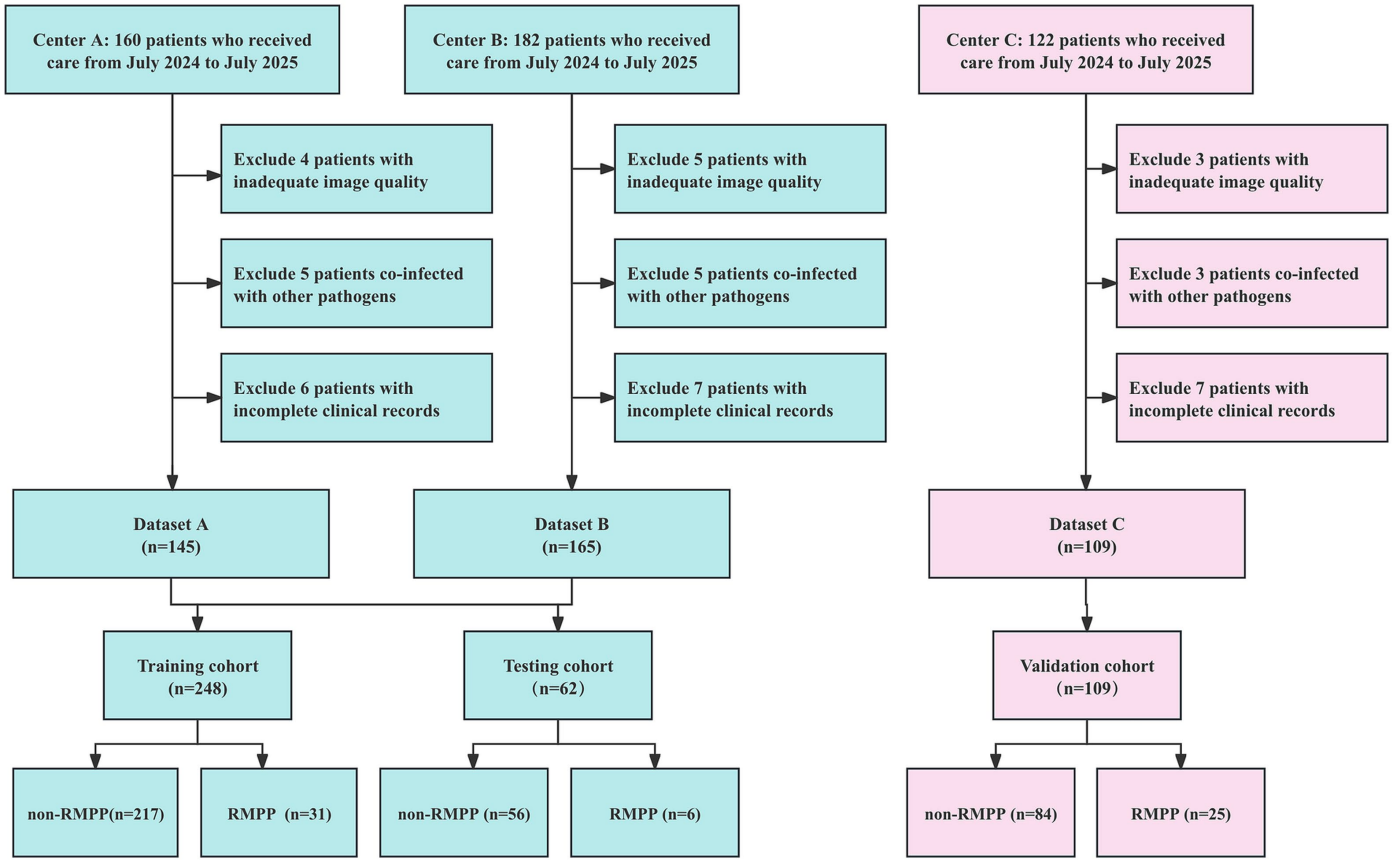
Flowchart illustrating participant selection based on inclusion and exclusion criteria in a multicenter cohort study. Non-RMPP, non-refractory *Mycoplasma pneumoniae* pneumonia; RMPP, refractory *Mycoplasma pneumoniae* pneumonia.

In total, 419 children with MPP were included, comprising 248 patients in the training cohort (non-RMPP, *n* = 217; RMPP, *n* = 31), 62 in the testing cohort (non-RMPP, *n* = 56; RMPP, *n* = 6), and 109 in the external validation cohort (non-RMPP, *n* = 84; RMPP, *n* = 25). The detailed inclusion and exclusion process is shown in Figure 2.

Significant inter-cohort differences were observed for CK-MB (*p* < 0.001), LDH (*p* < 0.001), APTT (*p* < 0.001), D-dimer (*p* < 0.001), type of fever (*p* < 0.001), lobar atelectasis (*p* = 0.007), consolidation pattern (*p* < 0.001), consolidation mixed with ground-glass opacity (*p* < 0.001), air bronchogram sign (*p* < 0.001), interlobular septal thickening (*p* < 0.001), fiber cord (*p* = 0.011), and number of lobes involved (*p* < 0.001, Table 1).

**Table 1:** Comparison of clinical and radiological characteristics between MPP and RMPP groups in training, testing and validation cohorts.

Characteristics	Training cohort (*n* = 248)			Testing cohort (*n* = 62)			Validation cohort (*n* = 109)			Overall (*n* = 419)
	Non-MPP (*n* = 217)	RMPP (*n* = 31)	*p*-intra value	Non-MPP (*n* = 56)	RMPP (*n* = 6)	*p*-intra value	Non-MPP (*n* = 84)	RMPP(*n* = 25)	*p*-intra value	*p*-inter value
Male	107 (49.309)	18 (58.065)	0.362	24 (42.857)	5 (83.333)	0.089	46 (54.762)	10 (40.000)	0.195	0.838
Age, years	7.000 [5.000, 9.000]	7.000 [6.000, 9.000]	0.266	7.000 [5.000, 9.000]	5.000 [3.500, 5.750]	0.080	7.000 [4.000, 11.000]	7.000 [4.000, 8.000]	0.704	0.939
WBC, ×10^9^/L	7.790 [6.270, 9.740]	8.100 [6.515, 9.850]	0.624	7.755 [6.543, 10.340]	6.985 [5.947, 7.782]	0.335	7.985 [5.178, 12.037]	9.440 [6.480, 12.550]	0.451	0.711
NEUT, ×10^9^/L	4.710 [3.510, 6.400]	5.200 [3.735, 6.685]	0.622	5.070 [3.562, 7.253]	4.220 [3.520, 4.718]	0.239	4.210 [2.448, 7.113]	4.920 [3.340, 6.680]	0.517	0.377
PLT, ×10^9^/L	284.000 [234.000, 369.000]	300.000 [252.000, 329.000]	0.912	275.500 [224.750, 347.500]	247.500 [238.000, 300.500]	0.896	284.500 [231.500, 372.250]	343.000 [273.000, 387.000]	0.070	0.263
CK-MB, ng/mL	2.330 [1.850, 2.770]	2.280 [1.805, 3.080]	0.508	2.430 [2.040, 2.820]	2.615 [1.875, 3.160]	0.840	0.700 [0.400, 1.200]	0.800 [0.600, 1.100]	0.302	<0.001
LDH, U/L	285.000 [246.000, 342.000]	275.000 [211.500, 331.500]	0.086	266.000 [227.000, 314.500]	302.000 [290.250, 361.750]	0.069	245.500 [182.750, 322.000]	246.000 [229.000, 277.000]	0.535	<0.001
APTT, s	33.400 [31.000, 35.800]	33.600 [32.050, 36.250]	0.594	32.950 [31.000, 34.250]	31.100 [30.525, 33.475]	0.422	31.700 [29.950, 34.025]	32.300 [28.200, 34.500]	0.629	<0.001
FIB, g/L	3.910 [3.450, 4.290]	3.760 [3.470, 4.150]	0.555	3.885 [3.500, 4.232]	3.860 [3.810, 3.947]	0.788	3.825 [2.968, 4.628]	4.360 [3.390, 4.960]	0.070	0.813
D-dimer, ng/mL	0.253 [0.166, 0.388]	0.190 [0.126, 0.453]	0.381	0.229 [0.138, 0.352]	0.366 [0.185, 0.538]	0.453	197.000 [137.000, 249.750]	312.000 [137.000, 470.000]	0.068	<0.001
CRP, mg/L	7.480 [2.420, 17.240]	6.530 [1.935, 19.965]	0.793	6.375 [2.935, 12.473]	18.155 [4.673, 48.460]	0.528	4.660 [1.038, 14.617]	13.300 [5.970, 25.870]	0.022	0.405
NLR	2.288 [1.572, 3.272]	2.091 [1.609, 3.673]	0.599	2.523 [1.730, 3.416]	1.788 [1.546, 2.129]	0.119	1.865 [0.981, 3.849]	1.550 [0.992, 3.572]	0.832	0.109
PLR	141.401 [102.913, 175.635]	144.262 [110.562, 167.210]	0.885	137.626 [108.270, 176.588]	126.796 [110.392, 134.495]	0.203	125.011 [89.497, 192.749]	144.615 [92.857, 179.021]	0.694	0.707
SII, ×10^9^/L	635.364 [403.284, 997.946]	646.535 [471.815, 944.065]	0.764	665.158 [482.625, 1141.411]	489.697 [403.683, 543.583]	0.103	583.958 [314.405, 1101.063]	531.807 [406.000, 939.512]	0.705	0.361
Type of fever			0.180			0.064			0.009	<0.001
Absent	10 (4.608)	0 (0.000)		3 (5.357)	0 (0.000)		31 (36.905)	1 (4.000)		
Low-grade	75 (34.562)	16 (51.613)		25 (44.643)	0 (0.000)		5 (5.952)	1 (4.000)		
Mid-grade	129 (59.447)	15 (48.387)		28 (50.000)	6 (100.000)		23 (27.381)	9 (36.000)		
Hyperpyrexia	3 (1.382)	0 (0.000)		0 (0.000)	0 (0.000)		25 (29.762)	14 (56.000)		
Lobar atelectasis	32 (14.747)	8 (25.806)	0.117	6 (10.714)	0 (0.000)	1.000	3 (3.571)	2 (8.000)	0.323	0.007
Consolidation pattern			0.404			0.229			0.046	<0.001
Absent	29 (13.364)	2 (6.452)		10 (17.857)	0 (0.000)		36 (42.857)	4 (16.000)		
Patchy	61 (28.111)	6 (19.355)		16 (28.571)	0 (0.000)		18 (21.429)	5 (20.000)		
Segmental	57 (26.267)	10 (32.258)		15 (26.786)	3 (50.000)		16 (19.048)	10 (40.000)		
Wedge-shaped	70 (32.258)	13 (41.935)		15 (26.786)	3 (50.000)		14 (16.667)	6 (24.000)		
Consolidation mixed GGO	131 (60.369)	19 (61.290)	0.922	32 (57.143)	4 (66.667)	1.000	26 (30.952)	14 (56.000)	0.023	<0.001
Adjacent pleura thickening	11 (5.069)	4 (12.903)	0.101	1 (1.786)	0 (0.000)	1.000	1 (1.190)	4 (16.000)	0.010	0.336
Pleural effusion			0.076			0.082			1.000	0.544
Absent	206 (94.931)	26 (83.871)		51 (91.071)	6 (100.000)		81 (96.429)	25 (100.000)		
Mild	7 (3.226)	4 (12.903)		4 (7.143)	0 (0.000)		1 (1.190)	0 (0.000)		
Moderate	2 (0.922)	1 (3.226)		0 (0.000)	0 (0.000)		1 (1.190)	0 (0.000)		
Severe	2 (0.922)	0 (0.000)		1 (1.786)	0 (0.000)		1 (1.190)	0 (0.000)		
Mediastinal enlargement of lymph nodes	11 (5.069)	1 (3.226)	1.000	5 (8.929)	0 (0.000)	1.000	1 (1.190)	1 (4.000)	0.408	0.149
Air bronchogram sign	151 (69.585)	25 (80.645)	0.204	34 (60.714)	6 (100.000)	0.081	26 (30.952)	16 (64.000)	0.003	<0.001
Interlobular septal thickening	67 (30.876)	13 (41.935)	0.218	15 (26.786)	2 (33.333)	0.662	6 (7.143)	2 (8.000)	1.000	<0.001
Reticular pattern	26 (11.982)	3 (9.677)	1.000	9 (16.071)	1 (16.667)	1.000	6 (7.143)	2 (8.000)	1.000	0.201
Fiber cord	40 (18.519)	4 (12.903)	0.445	13 (23.214)	2 (33.333)	0.626	24 (28.571)	11 (44.000)	0.147	0.011
Number of lobes involved			0.366			0.373			0.568	<0.001
1	78 (35.945)	16 (51.613)		19 (33.929)	2 (33.333)		57 (67.857)	16 (64.000)		
2	59 (27.189)	4 (12.903)		12 (21.429)	3 (50.000)		14 (16.667)	4 (16.000)		
3	38 (17.512)	6 (19.355)		11 (19.643)	0 (0.000)		4 (4.762)	1 (4.000)		
4	13 (5.991)	2 (6.452)		9 (16.071)	0 (0.000)		3 (3.571)	2 (8.000)		
5	29 (13.364)	3 (9.677)		5 (8.929)	1 (16.667)		6 (7.143)	2 (8.000)		

*p*-intra value is the result of univariate analyses between the MPP and RMPP groups; *p*-inter value represents the comparisons of characteristics between training, testing and validation cohorts.

MPP, *Mycoplasma pneumoniae* pneumonia; RMPP, refractory *Mycoplasma pneumoniae* pneumonia; WBC, white blood cell; NEUT, neutrophil count; PLT, platelet count; CK-MB, creatine kinase-MB; LDH, lactate dehydrogenase; APTT, activated partial thromboplastin time; FIB, fibrinogen; CRP, C-reactive protein; NLR, neutrophil-to-lymphocyte ratio; PLR, platelet-to-lymphocyte ratio; SII, systemic immune-inflammation index; GGO, ground-glass opacity.

### Variables associated with RMPP in the training dataset

In univariable logistic regression, higher inflammatory and injury-related indices were associated with RMPP, including WBC (OR = 1.149, *p* = 0.001), NEUT (OR = 1.221, *p* < 0.001), LDH (OR = 1.005, *p* < 0.001), CRP (OR = 1.034, *p* < 0.001), and NLR (OR = 1.263, *p* = 0.001). SII was also associated with RMPP (OR = 1.000, *p* = 0.020).

Among CT variables, lobar atelectasis was associated with RMPP (OR = 2.891, *p* = 0.001). Using absent consolidation as the reference, patchy (OR = 6.418, *p* = 0.001), segmental (OR = 4.306, *p* = 0.011), and wedge-shaped consolidation (OR = 8.716, *p* < 0.001) were associated with higher odds of RMPP. Pleural effusion (OR = 4.566, *p* = 0.002) and air bronchogram sign (OR = 1.798, *p* = 0.029) were also significant. For the number of lobes involved (reference: 1 lobe), involvement of 5 lobes was associated with RMPP (OR = 2.625, *p* = 0.035) (see Table 2).

**Table 2:** Univariate logistic regression analyses for selecting clinical and radiological features in the training cohort.

Predictor	Coefficient	OR (95% CI)	*p*-value
Male	−0.076	0.927 (0.584, 1.471)	0.747
Age	0.030	1.030 (0.799, 1.328)	0.819
Type of fever			
Low-grade	0.946	2.576 (0.543, 12.215)	0.233
Mid-grade	1.341	3.824 (0.823, 17.765)	0.087
Hyperpyrexia	2.398	11.000 (0.646, 187.176)	0.097
WBC	0.139	1.149 (1.062, 1.243)	0.001
NEUT	0.200	1.221 (1.104, 1.350)	<0.001
PLT	0.000	1.000 (0.998, 1.001)	0.664
LDH	0.005	1.005 (1.003, 1.008)	<0.001
APTT	−0.005	0.995 (0.937, 1.056)	0.857
FIB	0.292	1.340 (0.953, 1.883)	0.092
CRP	0.033	1.034 (1.019, 1.049)	<0.001
NLR	0.233	1.263 (1.100, 1.450)	0.001
PLR	0.000	1.000 (0.998, 1.002)	0.957
SII	0.000	1.000 (1.000, 1.001)	0.020
Lobar atelectasis	1.062	2.891 (1.524, 5.486)	0.001
Consolidation pattern			
Patchy	1.859	6.418 (2.093, 19.680)	0.001
Segmental	1.460	4.306 (1.394, 13.304)	0.011
Wedge-shaped	2.165	8.716 (2.893, 26.260)	<0.001
Consolidation mixed GGO	0.447	1.564 (0.967, 2.529)	0.068
Adjacent pleura thickening	0.573	1.774 (0.647, 4.862)	0.265
Pleural effusion	1.519	4.566 (1.734–12.024)	0.002
Mediastinal enlargement of lymph nodes	0.450	1.568 (0.588, 4.186)	0.369
Air bronchogram sign	0.587	1.798 (1.061, 3.046)	0.029
Interlobular septal thickening	−0.108	0.897 (0.544, 1.481)	0.671
Reticular pattern	−0.172	0.842 (0.414, 1.712)	0.634
Fiber cord	−0.432	0.649 (0.350, 1.205)	0.171
Number of lobes involved			
2	−0.007	0.993 (0.543, 1.816)	0.981
3	−0.262	0.769 (0.383, 1.544)	0.461
4	0.417	1.518 (0.720, 3.200)	0.273
5	0.965	2.625 (1.070, 6.441)	0.035

OR, odds ratio; CI, confidence interval; WBC, white blood cell; NEUT, neutrophil count; PLT, platelet count; LDH, lactate dehydrogenase; APTT, activated partial thromboplastin time; FIB, fibrinogen; CRP, C-reactive protein; NLR, neutrophil-to-lymphocyte ratio; PLR, platelet-to-lymphocyte ratio; SII, systemic immune-inflammation index.

### Diagnostic performance of different models for predicting RMPP

**Figure 3:**
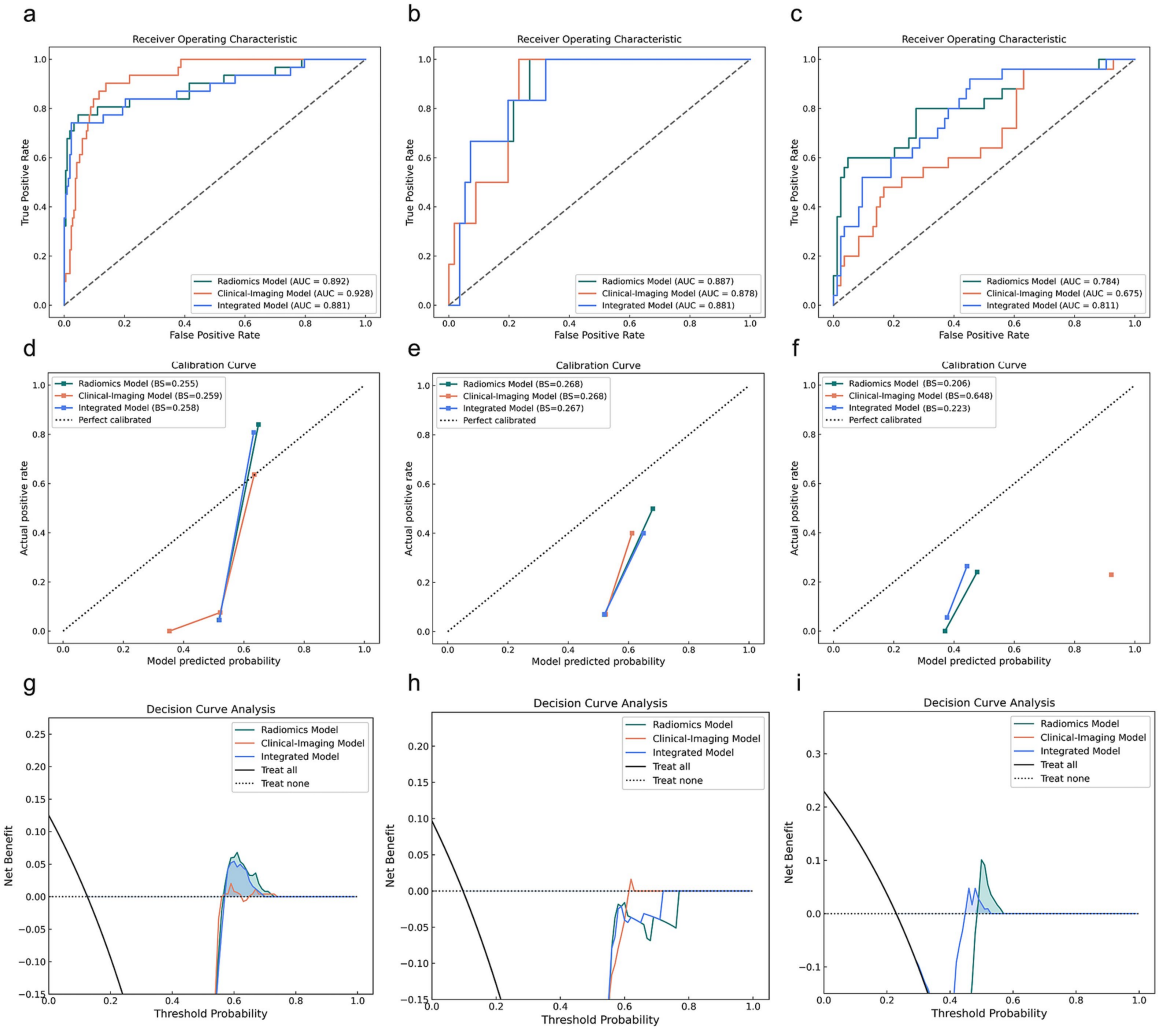
Predictive performance of the clinical-image, radiomics, and integrated models for RMPP stratification across the training, testing, and validation cohorts. **(a–c)** Receiver operating characteristic curves, **(d–f)** calibration curves, and **(g–i)** decision curve analyses for the training (left), testing (middle), and validation (right) cohorts.

Figure 3 presents the ROC curves, calibration curves, and decision curves for the three predictive models used in severity stratification.

In the validation cohort, the radiomics model achieved an AUC of 0.784 (95% CI, 0.683–0.885), accuracy of 0.789, sensitivity of 0.160, and specificity of 0.976. The clinical-imaging model achieved an AUC of 0.675 (95% CI, 0.603–0.833), accuracy of 0.229, sensitivity of 1.000, and specificity of 0.000. The integrated model achieved an AUC of 0.811 (95% CI, 0.704–0.917), accuracy of 0.872, sensitivity of 0.600, and specificity of 0.952 (see Table 3).

**Table 3:** Diagnostic performance of different models for predicting RMPP.

Model	AUC (95% CI)	Accuracy	*F*_1_-score	Precision	Sensitivity	Specificity
Training cohort						
Radiomics model	0.892 (0.814–0.970)	0.762	0.469	0.325	0.839	0.751
Clinical-imaging model	0.928 (0.888–0.967)	0.867	0.621	0.482	0.871	0.866
Integrated model	0.881 (0.799–0.963)	0.742	0.448	0.306	0.839	0.728
Testing cohort						
Radiomics model	0.887 (0.786–0.988)	0.758	0.400	0.263	0.833	0.750
Clinical-imaging model	0.878 (0.776–0.979)	0.790	0.435	0.294	0.833	0.786
Integrated model	0.881 (0.770–0.991)	0.694	0.345	0.217	0.833	0.679
Validation cohort						
Radiomics model	0.784 (0.683–0.885)	0.789	0.258	0.667	0.160	0.976
Clinical-imaging model	0.675 (0.603–0.833)	0.229	0.373	0.229	1.000	0.000
Integrated model	0.811 (0.704–0.917)	0.872	0.682	0.790	0.600	0.952

MPP, *Mycoplasma pneumoniae* pneumonia; AUC, area under the receiver operating characteristic curve; CI, confidence interval.

### Diagnostic performance of different models in RMPP

Pairwise DeLong tests showed no significant AUC differences between models in validation cohort (all *p* > 0.05). McNemar tests indicated significant differences in classification results between selected model pairs in the external validation cohort, including radiomics vs. clinical-imaging (*p* = 0.001), radiomics vs. integrated (*p* = 0.013), and clinical-imaging vs. integrated (*p* < 0.001).

Reclassification performance differed across model pairs. In the validation cohort, the integrated model yielded a significantly higher NRI than the radiomics model (*p* < 0.001) and the clinical–imaging model (*p* < 0.001), whereas the NRI difference between the radiomics and clinical–imaging models was not significant (*p* = 0.070) (see Table 4).

**Table 4:** Comparison of different models in predicting RMPP.

Method	DeLong test		McNemar	NRI		IDI	
	*Z* value	*p*-value	*p*-value	*Z* value	*p*-value	*Z* value	*p*-value
Training cohort							
Radiomics vs. clinical-imaging	0.763	0.446	0.405	−0.783	0.434	−1.503	0.133
Radiomics vs. integrated	1.070	0.285	0.219	−0.828	0.408	−2.679	0.007
Clinical-imaging vs. integrated	0.964	0.335	<0.001	0.150	0.881	0.685	0.494
Testing cohort							
Radiomics vs. clinical-imaging	0.109	0.914	<0.001	−0.243	0.808	−0.300	0.764
Radiomics vs. integrated	0.414	0.679	0.063	0.000	1.000	0.619	0.536
Clinical-imaging vs. integrated	0.034	0.973	<0.001	0.218	0.827	0.404	0.686
Validation cohort							
Radiomics vs. clinical-imaging	1.234	0.217	0.001	1.811	0.070	0.145	0.885
Radiomics vs. integrated	0.708	0.479	0.013	−4.135	<0.001	−1.275	0.202
Clinical-imaging vs. integrated	1.857	0.063	<0.001	−5.486	<0.001	−0.502	0.616

MPP, *Mycoplasma pneumoniae* pneumonia; NRI, net reclassification index; IDI, integrated discrimination improvement.

### SHAP analysis

**Figure 4:**
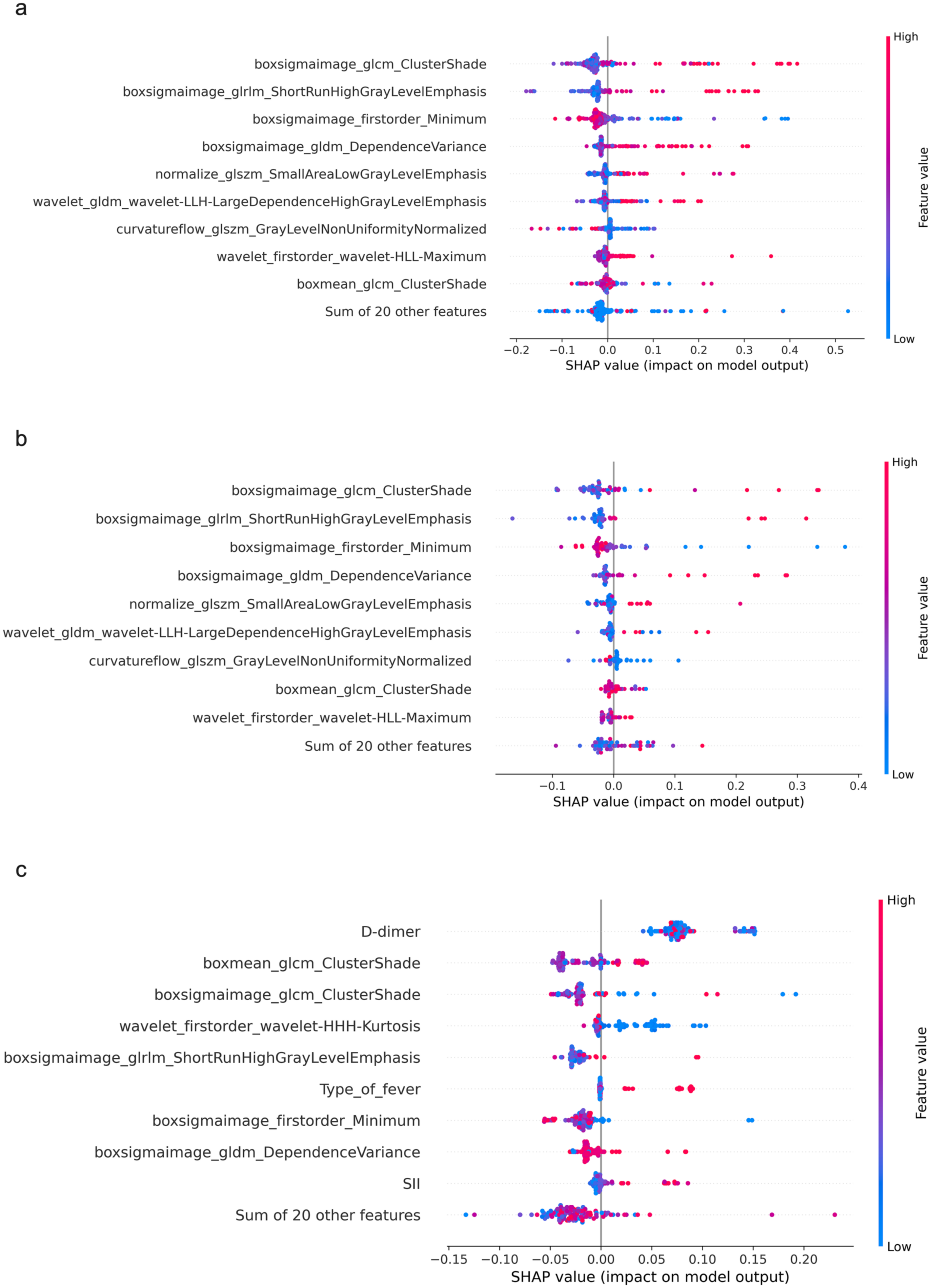
Global SHAP analysis of the integrated model. SHAP summary plots illustrate the global feature importance and their effects on model predictions in the training cohort **(a)**, testing cohort **(b)**, and validation cohort **(c)**.

In the external validation cohort (Figure 4), global SHAP analysis identified D-dimer, Type of fever, and SII as leading contributors, together with radiomics features including boxmean glcm ClusterShade, boxsigmaimage glcm ClusterShade, wavelet firstorder wavelet-HHH-Kurtosis, and boxsigmaimage glrlm ShortRunHighGrayLevelEmphasis.

**Figure 5:**
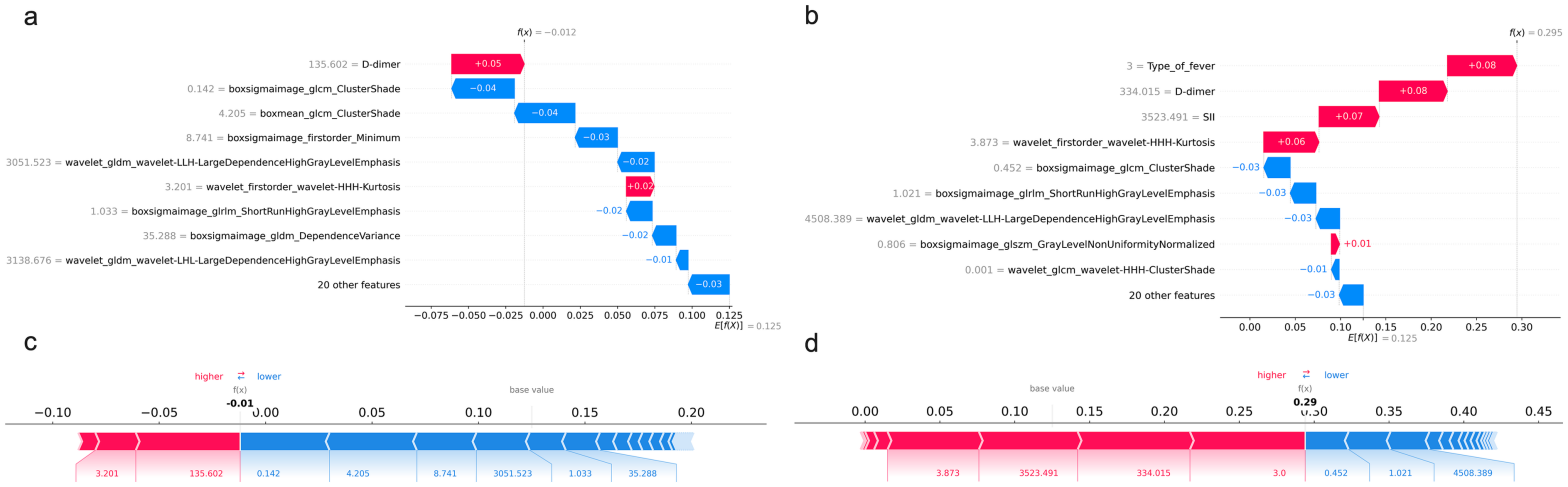
Case-level SHAP analysis of the integrated model for non-RMPP and RMPP cases. SHAP force plots illustrate the contribution of individual features to model predictions for representative non-RMPP cases **(a,c)** and RMPP cases **(b,d)**.

Local SHAP explanations (Figure 5) indicated that the non-RMPP cases (Figures 5a,c) reduced the prediction from the baseline *E* [*f*(*X*)] = 0.125 to *f*(*x*) = −0.012, while the RMPP cases (Figures 5b,d) increased it to *f*(*x*) = 0.295, with the largest positive contributions driven by type of fever, D-dimer, and SII.

## Discussion

This multicenter study demonstrates that early risk stratification for RMPP can be significantly enhanced by utilizing CT-based radiomics, especially when integrated with selected clinical and laboratory parameters. In the validation cohort, the integrated model achieved significantly superior reclassification compared to both alternative models.

Our findings demonstrate that the integrated model significantly outperformed single-modality approaches in predicting RMPP in children, as evidenced by its superior NRI in validation cohorts. This enhanced performance can be attributed to the capacity of radiomics to augment clinical data by capturing complementary biological information (16). The radiomics features incorporated in this study, such as wavelet_firstorder_wavelet-HHH-Kurtosis, represent higher-order texture information from lung tissue, capturing fine-scale variations in tissue density and structure (17). These features may correlate with tissue injury and parenchymal damage, both of which are prevalent in RMPP, characterized by more extensive and heterogeneous damage. Furthermore, boxsigmaimage_glrIm_Short Run High Gray Level Emphasis highlights regions of intense contrast within consolidated areas, potentially reflecting localized inflammation, edema, or fibrosis—hallmarks of RMPP’s progressive nature. boxsigmaimage_firstorder_Minimum and boxsigmaimage_gldm_DependenceVariance focus on the intensity and spatial dependency of pixel values in lung tissue, providing insights into consolidation heterogeneity and early indicators of pulmonary dysfunction associated with RMPP. These features, in synergy with clinical markers such as D-dimer and SII, enable the integrated model to provide a more granular, multifactorial risk assessment. This model reflects the pathophysiology of RMPP, involving complex interactions among inflammatory markers, coagulation abnormalities, and tissue damage that led to refractory disease. The low specificity of the clinical-imaging model in the external validation cohort primarily reflects inter-center heterogeneity in clinical and laboratory variables, underscoring the complementary value of radiomics features in improving generalizability.

Prior research on RMPP has emphasized that significantly higher levels of LDH, D-dimer, CRP, and NLR are observed in RMPP compared to common MPP (18–20), thereby supporting the directionality of the associations observed in this study. The univariable associations observed in the training dataset provide a coherent biological narrative: inflammatory and tissue-injury markers (WBC, NEUT, CRP, LDH, NLR, SII) were positively associated with RMPP, along with CT phenotypes indicating more extensive or obstructive parenchymal involvement (lobar atelectasis, pleural effusion, specific consolidation patterns, and broader lobar distribution). This pattern aligns with the prevailing understanding of RMPP as a phenotype driven not only by pathogen factors but also by a dysregulated host inflammatory response and downstream tissue damage (21).

The interpretability layer bolsters mechanistic plausibility, suggesting that systemic hyperinflammation and coagulation activation—specifically D-dimer, fever type, and SII—interact with heterogeneous intrapulmonary injury patterns (radiomics) to drive refractory trajectories (22). While the link between D-dimer and RMPP is well-established, the prominence of the SII as a top contributor offers novel insights. By integrating neutrophil, platelet, and lymphocyte counts, SII captures a pathogenic “perfect storm” of neutrophilic hyperinflammation, thrombotic potential, and immune exhaustion (23). This suggests that RMPP is driven by a complex interplay between systemic immune dysregulation and coagulation rather than a single pathway (24). In our model, these systemic markers likely act as a bridge to the structural damage captured by radiomics, reflecting how chaotic host responses manifest as dense, heterogeneous lung consolidation.

Radiologically, lobar atelectasis, consolidation and number of lobes involved represents dense inflammatory exudates within alveolar spaces, resulting in impaired gas exchange, reduced mucociliary clearance, and a local environment conducive to secondary infections and hypoxia-induced injury (25). This imaging feature aligns with the established pathophysiological understanding that alveolar inflammation and structural lung damage are key contributors to prolonged disease resolution (26, 27). The coexistence of consolidation with elevated D-dimer and SII in our cohort further underscores this mechanistic triad: vascular injury (reflected by D-dimer), neutrophil-dominated hyperinflammation (SII), and structural lung damage (consolidation) collectively perpetuate tissue hypoxia and repair delays. Clinically, these findings support early stratification of patients exhibiting consolidation patterns to guide targeted interventions (28), such as adjunctive corticosteroids to mitigate inflammation or proactive pulmonary rehabilitation to prevent fibrotic sequelae.

Conventional approaches often rely on semi-quantitative CT scoring or expert-defined features. For instance, a study which combined bronchoscopy-based bronchitis scores with CT scores, reported an AUC of 0.82, underscoring both the predictive value of extent-based imaging severity and the trade-off between sensitivity and specificity inherent in coarse scoring systems (29). Relative to such approaches, CT radiomics offers two key advances: (a) it captures sub-visual heterogeneity beyond lesion extent, and (b) it is more readily standardized and scalable than subjective scoring, although it remains sensitive to segmentation and acquisition variability. Recent AI-enabled imaging studies provide an informative benchmark. A study utilized automated AI-driven CT quantification of lesion burden alongside clinical variables, achieving high AUC (0.895) for a combined model in their setting (30). Compared to that work, the external AUC (0.811) in the present study is more modest; however, the design is multicenter with explicit external validation and demonstrates that incorporating texture-level radiomics can enhance reclassification robustness amid inter-site heterogeneity—an issue that single-center or internally validated studies may underestimate.

### Limitations

Although this research highlights the clinical promise of the integrated model, certain drawbacks should be noted. First, as a retrospective study, this design is inherently susceptible to selection bias and precludes the establishment of definitive causal relationships. Its generalizability may be limited in low-resource settings characterized by heterogeneous equipment and restricted biomarker availability. Second, the model relies on high-resolution chest CT, raising radiation exposure concerns in pediatric populations and restricting applicability in regions where CT is not routinely available or where radiation risk is a major constraint. Third, detailed pre-admission macrolide exposure and days from symptom onset to admission were not systematically documented owing to the retrospective design. This may have partially confounded RMPP outcome labeling and could slightly overestimate the performance of early prediction. Future prospective multicenter studies should record these variables to further strengthen model validity. Future studies should therefore prioritize prospective designs across diverse healthcare settings, including low-resource environments, and incorporate standardized imaging protocols and additional biomarkers to confirm clinical utility and support widespread implementation.

## Conclusion

In conclusion, CT-based radiomics offers a promising and interpretable approach for early identification of RMPP, particularly when combined with clinically relevant biomarkers. The integrated model may become practical tools to support timely, individualized decision-making in pediatric MPP care.

## Data Availability

The raw data supporting the conclusions of this article will be made available by the authors, without undue reservation.
